# Supplementary material for: rAAV Engineering for Capsid-Protein Enzyme Insertions and Mosaicism Reveals Resilience to Mutational, Structural and Thermal Perturbations
Source: Int J Mol Sci. 2019 Nov 14;20(22):5702. doi: 10.3390/ijms20225702 (PMC6887778; doi:10.3390/ijms20225702)
Supplement: Supplementary file 1 [file ijms-20-05702-s001.pdf]

Supplementary information

rAAV engineering for capsid-protein enzyme insertions and mosaicism reveals resilience to mutational, structural and thermal perturbations

Rebecca C. Feiner[1†], Julian Teschner[1†], Kathrin E. Teschner[1], Marco T. Radukic[1], Tobias Baumann[2], Sven Hagen[3], Yvonne Hannappel[4], Niklas Biere[5], Dario Anselmetti[5], Katja M. Arndt[6], Kristian M. Müller[1*]

Contents

1 Supplementary Information—Methods ... 1
1.1 Method S1: Plasmid construction ... 1
1.1.1 Plasmids constructed in this work ... 1
1.1.2 Plasmids previously generated ... 2
1.2 Method S2: ITR Sanger sequencing ... 4
2 Supplementary Information—Figures ... 5
2.1 Figure S1 ... 5
2.2 Figure S2 ... 6
2.3 Figure S3 ... 7
2.4 Figure S4 ... 8
2.5 Figure S5 ... 9
2.6 Figure S6 ... 9
2.7 Figure S7 ... 10
2.8 Figure S8 ... 10
2.9 Figure S9 ... 11
3 Supplementary Information—Tables ... 12
3.1 Table S1 ... 12
3.2 Table S2 ... 12
3.3 Table S3 ... 13
4 Cloning strategy ... 13
4.1 Cloning of loop modifications in pZMB0216 ... 13
4.2 Cloning of N-terminal VP2 modifications for rAAV2 ... 14

1 Supplementary Information—Methods

1.1 Method S1: Plasmid construction

1.1.1 Plasmids constructed in this work

pZMB0522_ITR_EXS_CMV_mVenus_hGHpA. This is the streamlined version of the rAAV ITR plasmid. To allow BioBrick compatible integration of expression casettes into the ITR plasmid, the region in-between the ITRs of vector pGolden-AAV (Addgene plasmid # 51424 was a gift from Yonglun Luo [47]) was changed and EcoRI, XbaI as prefix and SpeI as suffix sites were integrated by hybridized oligonucleotides through the unique BsmFI and AgeI sites. The backbone of this new

generated RFC[10]-compatible ITR plasmid was digested with PstI and ligated with PCR-amplified pUC19 backbone (pUC19_PstI_for 5′-CTGCAGAAAA GGCCAGCAAA AGGC and pUC19_PstI_rev 5′-CTGCAGGCAC TTTTCGGGGA AATG) yielding pUC19_ITR_EXS. In the last step, the previously assembled BioBrick CMV_mVenus_hGHpA was cloned via EcoRI and SpeI into pUC19_ITR_EXS to generate pZMB0522_ITR_EXS_CMV_mVenus_hGHpA. This plasmid can be used to test GOI expression and at the same time allows integration of other genes. Note that XbaI and SpeI generate compatible cohesive ends. Facilitating new assemblies, a BioBrick CMV promoter (pZMB0143-CMV) and BioBrick hGHpA (pZMB0135_hGHpA) are also available.

pZMB0217_Rep_VP123_453_587wtGG_p5tataless, pZMB0218_Rep_VP123_453_587wtGGSG_p5tataless, pZMB0219_Rep_VP123_453_587wt2xGGSG_p5tataless, pZMB0220_001_Rep_VP123_453_587wt4xGGSG_p5tataless. These plasmids were constructed based on pZMB0216_Rep_VP123_453_587wt_p5tataless. A sequence coding for a Gly-Gly linker was integrated in position 587 by hybridization of oligonucleotides as described in Figure S11 upon digestion with BamHI and PvuII. The cloning strategy for the other GGSG linker constructs is similar to this one.

pZMB0221_VP123_453_587wtbla_p5tataless. This plasmid was constructed based on iGEM plasmid BBa_K404250. Amongst others BBa_K404250 codes for a thermostabilised variant of β-lactamase which was amplified by PCR using BamHI_wt_bla-for 5′-AAAGGATCCG TATCTACCAA CCTCCAGAGA GGCAACCACC CAGAAACGCT GGCGAAAG and PvuII_wt_bla-rev 5′-AAACAGCTGT AGCTGCTTGT CTCCAATGCT TAATCAGTGA GGCACC primers. The BamHI and PvuII digested PCR product was inserted in 587 position of pZMB0216_Rep_VP123_453_587wt_p5tataless by standard cloning techniques.

pZMB0315_CMV_Kozak_VP2_453_587wtHis. This vector was constructed in two steps starting from pZMB0156_VP23_453_587wt. After RFC[10] integration of the CMV promoter, the VP3 initiation codon was mutated from ATG (Met) to ATC (Ile) using site-directed mutagenesis primers SDM_VP3ko_for 5′-CTAATACGAT CGCTACAGGC AGTGGC and SDM_VP3ko_rev 5′-CCTGTAGCGA TCGTATTAGT TCCCAGAC. In the second step, a strong Kozak sequence was introduced via PCR primers XbaI_CMV-for 5′-CTTCTAGAGC GATGTACGGG and CMV_Kozak_NgoMIV-rev 5′-ATAATGCCGG CCATGGTGGC CTAGTAATTT CGATAAGCCA GTAAG and recloning of the fragment. The HisTag in 587 position was integrated by hybridization of oligonucleotide as described in Figure S11.

pZMB0577_001_pCMV_Kozak_VP2_453_587wtbla_p5tataless. This plasmid was constructed by PCR amplification of β-lactamase was using plasmid BBa_K404250 as template and primers BamHI_wt_bla-for 5′-AAAGGATCCG TATCTACCAA CCTCCAGAGA GGCAACCACC CAGAAACGCT GGCGAAAG and PvuII_wt_bla-rev 5′-AAACAGCTGT AGCTGCTTGT CTCCAATGCT TAATCAGTGA GGCACC, digestion of the PCR product with BamHI and PvuII and cloning the fragment in likewise opened pZMB0315_CMV_Kozak_VP2_453_587wtHis.

pZMB0601_001_pCMV_Kozak_VP2_453_587wt4xGGSG. This plasmid was constructed based on pZMB0315_CMV_Kozak_VP2_453_587wtHis. The sequence for wt4xGGSG was introduced into the 587 position by hybridization of oligonucleotides as described in Figure S11 upon digestion with BamHI and PvuII.

1.1.2 Plasmids previously generated

pZMB0216_Rep_VP123_453_587wt_p5tataless. Base pair numbering given here refers to the start codon of the RepCap coding region. Starting with the pAAV-RC plasmid (GenBank: AF369963.1) silent mutations of two PstI restriction sites (nucleotide substitutions G177C and G3940C) in the Rep coding region were introduced by site-directed mutagenesis (QuikChange II Site-Directed Mutagenesis Kit, Agilent Technologies). Two EcoRI sites (substitutions A1449G and T1668C) and a PstI site (substitution C1641T) were removed by cloning a synthesized DNA fragment (GeneArt, Darmstadt, Germany) into rep via BstEII/SwaI. To enable restriction-based modification of the 453 or 587 loop coding sequences, the unique restriction sites SspI/SalI (G3202A, C3205T, C3206T; T3254A, C3255G, A3256T, A3257C, G3259A) and BamHI/PvuII (T3613A, T3616C; C3658A, A3661T)

were introduced adjacent to the 453 and 587 loop of the viral capsid protein (VP), respectively. This was achieved by cloning a second synthesized DNA fragment (GeneArt) into the respective region via XcmI and BsiWI. BamHI (C729T) and SalI (C1110G) within the Rep coding region were removed by silent site-directed mutagenesis. The final RepCap construct was cloned in pSB1C3_001 via PCR using the primer Prefix_Rep68_78-ex (5′-GGAATTCGCG GCCGCTTCTA GATGGCGGGG TTTTACGAGA TTGTGATTAA G) and Suffix VP123ex RFC_25 5′-GCTACTAGTA TTAACCGGTG TAGTTAATGA TTAACCCGCC ATGCTACTTA TC) yielding pSB1C3_001_Rep_VP123_453_ 587wt. The promoter p5 TATA-less was converted into a BioBrick using p5_primer_for 5′-GCTCTAGAGG GAGGGGTGGA GTCGTGACGT G and p5_primer_rev 5′-TTCTGCAGCGG CCGCTACTAG TAGTTCAAAC CTCCCGCTTC AAAATGG and was cloned into pSB1C3_001_Rep_VP123_453_587wt via RFC[10] to generate the final construct pZMB0216_Rep_VP123_453_587wt_p5tataless.

pZMB0600_Rep_VP13_453_587ko_p5tataless. This plasmid was obtained by silent site-directed mutagenesis of the ACG initiation codon of VP2 in pZMB0216_Rep_VP123_453_587wt_p5tataless with the primers: VP2-ko_for 5′-GGTTGAGGAACCTGTTAAGACCGCTCCGGGAAAAAAGAGG and VP2-ko_rev 5′-CCTCTTTTTTCCCGGAGCGGTCTTAACAGGTTCCTCAACC to ACC.

pZMB0091_CMV_DARPinE01_mli_VP23 _453_587koHis. This vector is the product of two BioBrick cloning steps. First DARPinE01 with a GGSGGGSG linker sequence was cloned via RFC[25] into pZMB0156_VP23_453_587wt and then the CMV promoter from pZMB0143_CMV was added via RFC[10]. The HSPGko and the His-tag were integrated into the 587 loop region via hybridization of oligonucleotides as described in **Figure S11**.

pZMB0246_CMV_VP1up_NLS_mVenus_VP23_453_587koHis. This plasmid was generated by integrating the mVenus gene in pZMB0156_VP23_453_587wt fusing the VP1up_NLS part from pZMB0503_VP1up_NLS using the RFC[25] standard and adding the CMV promoter of pZMB0143_CMV. Finally, the 587 loop region was modified by hybridization of oligonucleotide coding for a His-Tag and the HSPGko as described in Figure S11.

pZMB0156_VP23_453_587wt. This vector is a BioBrick plasmid for subcloning purposes and was constructed by PCR amplification of the cap part from pZMB0216_Rep_VP123_453_587wt_p5tataless with the primers Prefix_VP2ex 5′-ATGGCCGGCG CTCCGGGAAA AAAGAGGCCG and Suffix_VP123ex_RFC_25 5′-GCTACTAGT ATTAACCGGT GTAGTTAATG ATTAACCCGC CATGCTACTT ATC followed by cloning the product into pSB1C3_001 via RFC[10] to generate pZMB0156_VP23_453_587wt. This plasmid serves for further N-terminal modifications. Thus, the VP3 initiation codon was not mutated. R585 and R588 (587ko) was introduced by hybridization of oligonucleotide as described in Figure S11.

pZMB0503_VP1up_NLS. A fragment of VP1 named VP1up was converted to a RFC[25] Biobrick by PCR amplification from pAAV-RC using the primers Prefix_VP1ex 5′-ATGGCCGGCG CTGCCGATGG TTATCTTCCA G and VP1up_Suffix_rev 5′-GGACTAGTA TTAACCGGTC GCCTTAACAG GTTCCTCAAC CAGG and cloning into pSB1C3. The NLS was generated in RFC[25] standard with the oligonucleotides NLS_for 5′-AATTCGCGGC CGCTTCTAGA TGGCCGGCCC TGCAAGAAAA AGATTGAATA CCGGTTAATA CTAGTAGCGG CCGCTGCA and NLS_rev 5′-GCGGCCGCT ACTAGTATTA ACCGGTATTC AATCTTTTTC TTGCAGGGCC GGCCATCTAG AAGCGGCCGC G and cloned downstream of the VP1up sequence.

pZMB0143_CMV. The CMV promoter was kindly provided by the iGEM team Ljubljana 2007 (BBa_I712004). A sequence alignment of this BioBrick with the CMV promoter used in pAAV-MCS shows slight differences.

pZMB0135_hGHpA. The human growth hormone polyadenylation signal (hGHpA) was converted into RFC[10] by PCR using pAAV-MCS as template and hGH_primer_for 5′-GCTCTAGACG GGTGGCATCC CTGTGAC and hGH_primer_rev 5′-GAACTGCAGC GGCCGCTACT AGTAAGGACAG GGAAGGGAGC AG and cloning the fragment in a pSB1C3 backbone.

1.2 Method S2: ITR Sanger sequencing

About 60 µg of ITR plasmid pZMB0522 were digested with 120 U MlyI in a total volume of 240 µl at 37 °C for 1.5 h and separated on an agarose gel. lITR and rITR fragments were isolated using NucleoSpin Gel and PCR Clean-up kit (MACHEREY-NAGEL). About 18 µg of each fragments were digested with 60 U BsaHI in a total volume of 120 µl at 37 °C for 2 h to split the sequence forming the major stem loop of the ITR sequence in half. Resulting fragments were separated on an agarose gel and 5′- and 3′-parts of the lITR and rITR were isolated. Sufficient material for several sequencing reactions was recovered. The obtained four DNA fragments were analysed by Sanger DNA-sequencing (Sequencing Core Facility, CeBiTec, Bielefeld, Germany). The sequencing primers were SEQ-lITR-5: 5′-GAAATGTTGA ATACTCATAC TCTTCC, SEQ-lITR-3: 5′-ATGAACTAAT GACCCCGTAA TTG, SEQ-rITR-5: 5′-CCTAATCTCA GGTGATCTACC and SEQ-rITR-3: 5′-AACGCCTGGT ATCTTTATAG TCC. Results are shown in Figure S2.

2 Supplementary Information—Figures

2.1 Figure S1

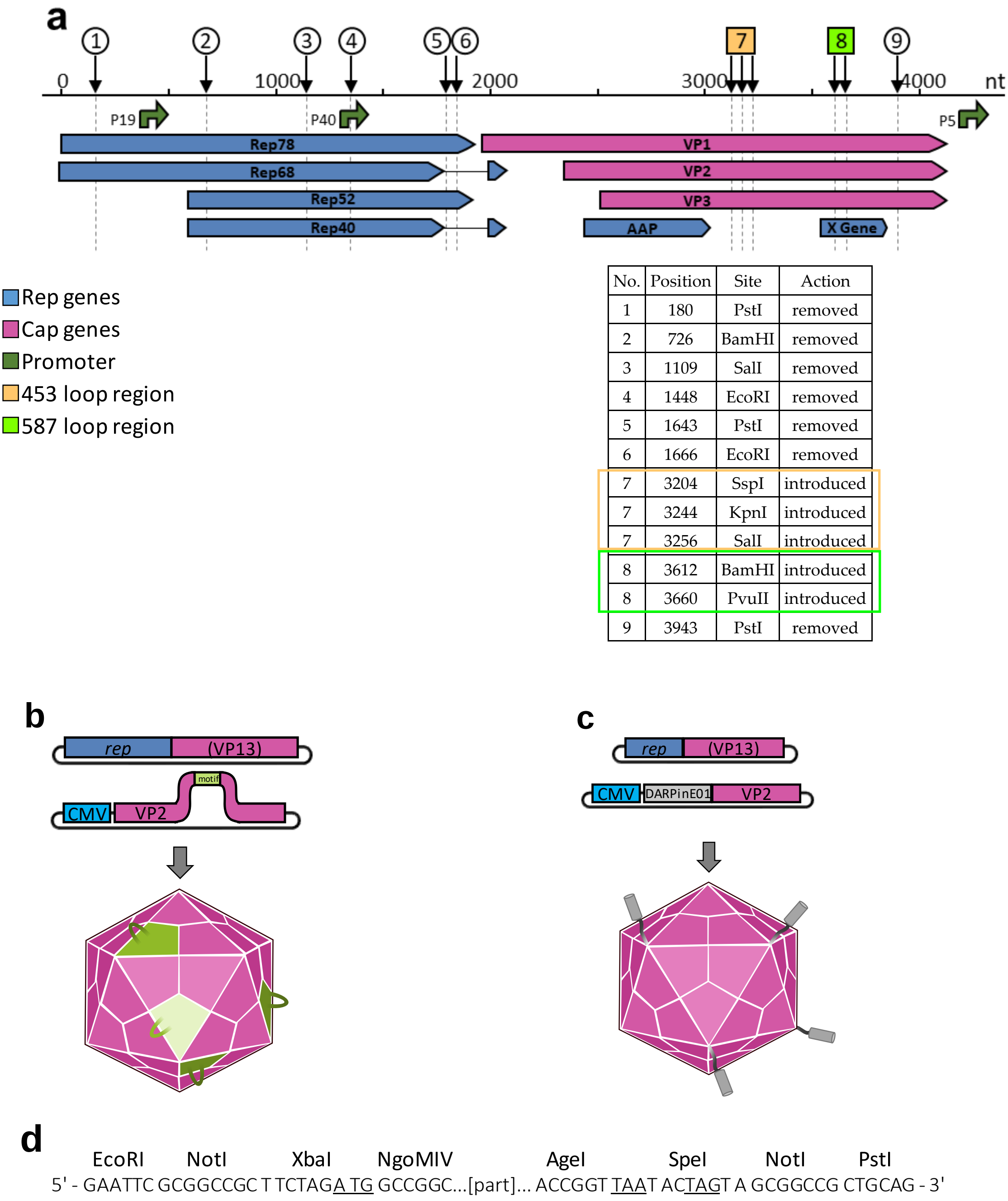

No.	Position	Site	Action
1	180	PstI	removed
2	726	BamHI	removed
3	1109	SalI	removed
4	1448	EcoRI	removed
5	1643	PstI	removed
6	1666	EcoRI	removed
7	3204	SspI	introduced
7	3244	KpnI	introduced
7	3256	SalI	introduced
8	3612	BamHI	introduced
8	3660	PvuII	introduced
9	3943	PstI	removed

Figure S1. Construction of the AAV2 RepCap plasmid (pZMB0216_Rep_VP123_453_587wt_p5tataless; Freiburg iGEM team 2010) and schematic overview of the rAAV plasmid production system. **a)** *Rep* and *cap* genes were integrated into the pSB1C3_001 backbone. Restriction sites not compatible with the RFC[10] standard were eliminated. Removal of restriction sites is marked with numbers and explained in the inset. For easy modification of 453 and 587 loop regions, the recognition sequences of SspI and SalI, as well as BamHI and PvuII were introduced. These allow for easy modification of the loop regions. Promoters are marked as green arrows. **b)** A forth plasmid allows for the production of mosaic rAAVs with e.g. modified 453 or 587 loop regions (green, e.g. His-tag) of the virion. Expressing a modified VP2 from a separate plasmid reduces the number of motifs on the capsid surface. **c)** Displaying proteins (grey, e.g. DARPinE01) on the capsid surface is also possible using a N-terminal fusion to VP2. **d)** Depiction of the prefix and suffix of the idempotent BioBrick RFC[25] cloning standard.

2.2 Figure S2

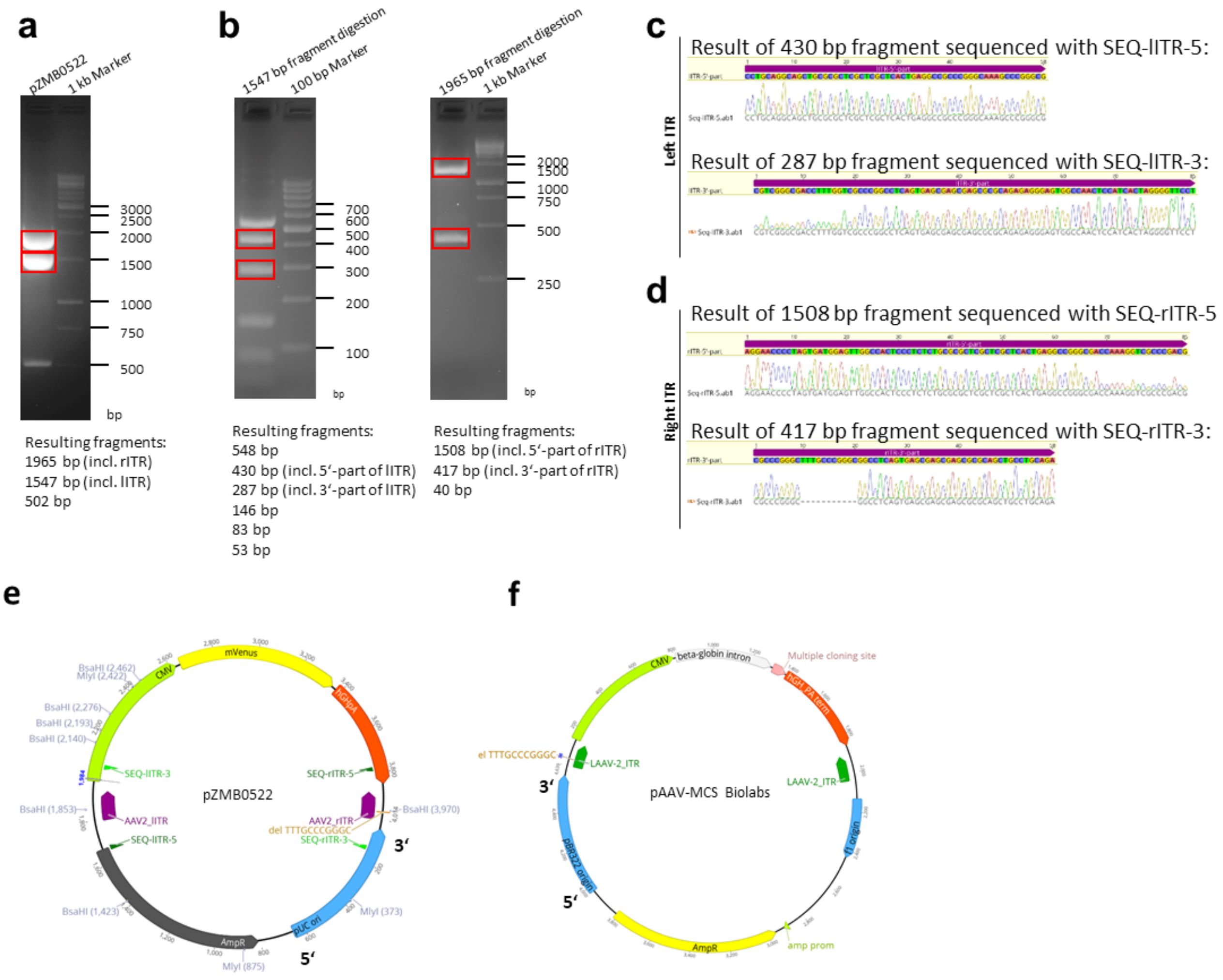

Figure S2. Preparation of fragments suitable for sequencing of ITRs**. a, b)** Agarose gel electrophoresis using a 1 % gel at a voltage of 120 V for 90 min. **a)** The restriction digest of pZMB0522 with MlyI results in three fragments and both ITRs are separated on an agarose gel as highlighted in red. **b)** The larger fragments are further digested with BsaHI to break up the T-shaped stem loop and to enable Sanger DNA-sequencing. **c, d)** Results of Sanger-DNA sequencing aligned to original ITR sequences. Fragments were sequenced with oligonucleotides given in the methods. **c)** The left ITR is completely intact. **d)** The 3′-part of rITR shows a deletion of 11 bp (5′-TTTGCCCGGGC-3′). **e)** Schematic overview of the ITR plasmid with used restriction sites. The 3′ end of the ori is orientated towards the right ITR. **f)** Overview of the commercially available pAAV-MCS from Cell Biolabs with highlighted left and right ITR. The 3′ end of the ori is oriented towards the left ITR which shows the 11 bp deletion. The distance between 3′ end of the ori and the ITR with deletions is 74bp and 85 bp, respectively.

We hypothesize that the distance of an ITR to the plasmid origin, the type of origin and the occurrence of Okazaki fragment processing sites may affect genetic ITR stability. In particular we assume that the *E. coli* PolI itself and the switch to PolII, which takes place shortly downstream of pMB1 type ori, contribute to the genetic instability.

Note that the original modular ITR plasmid was numbered and presented as most ITR plasmids (panel f) and the deletion carrying ITR was the left or 5′ ITR. In the new ITR plasmid (pZMB0522) we kept the classical pUC numbering and thus the deletion is formally now in the right or 3′ ITR.

2.3 Figure S3

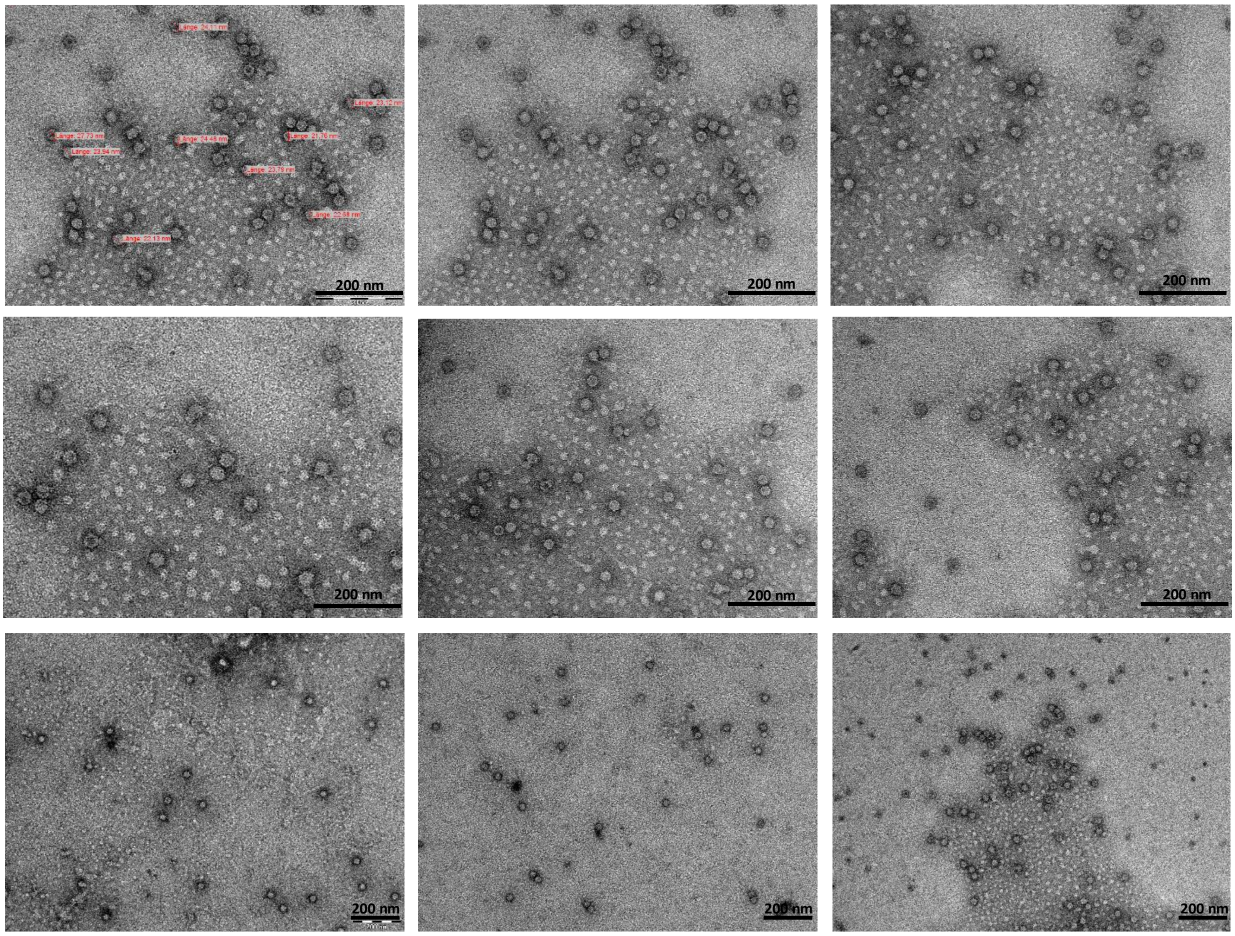

Figure S3. Transmission electron microscopy micrographs to calculate the full to empty capsid ratio of rAAV particles. An overview of images used for calculations is shown. Samples were applied to carbon-coated copper grids and stained with 2% uranyl acetate replacement stain (Science Services). rAAVs were visualized with a Philips CM100 (PW6021) with an acceleration voltage of 80 kV. Image analysis was performed using Soft Imaging viewer (Olympus). Scale bars indicate 200 nm. The first image shows diameter measurements to verify the diameter of rAAV particles.

2.4 Figure S4

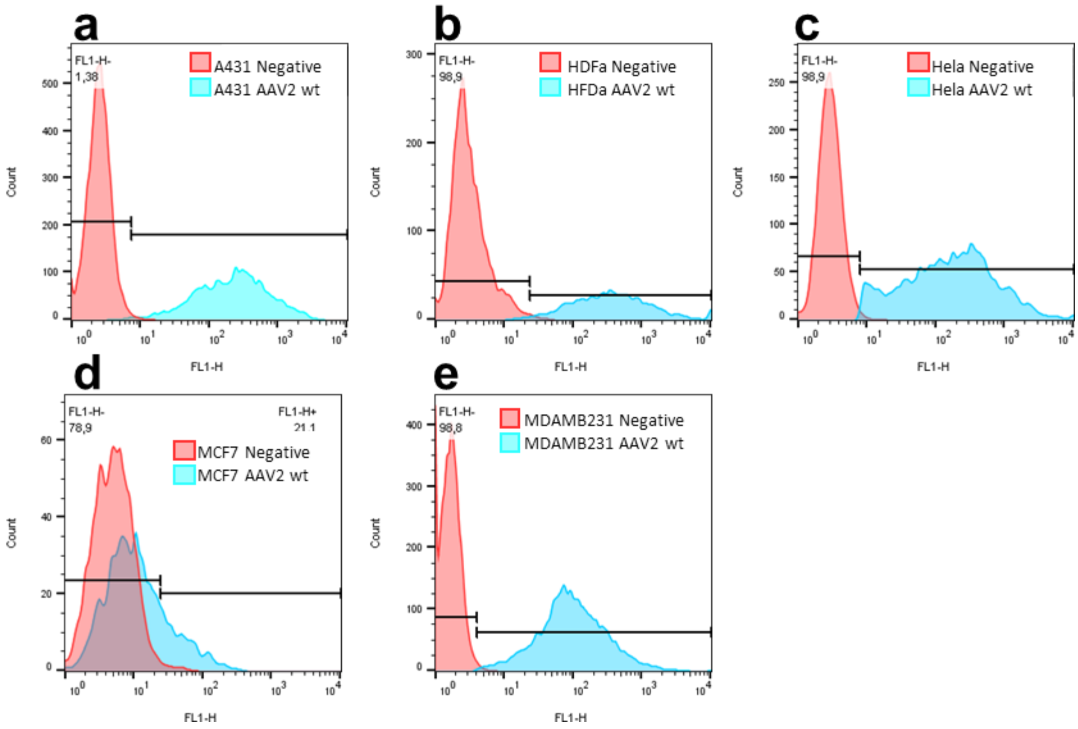

Figure S4. Flow cytometry data for rAAV variants in comparison to the negative buffer control. Cells were transduced with rAAVs at a MOI of 10,000 and analyzed via flow cytometry after incubation. Data analysis was performed using FlowJo. In a first step a population of live cells was gated. In a second step a gate of 1% false positive cells was selected in the sample of the negative control. This gate is visualized in each diagram.

2.5 Figure S5

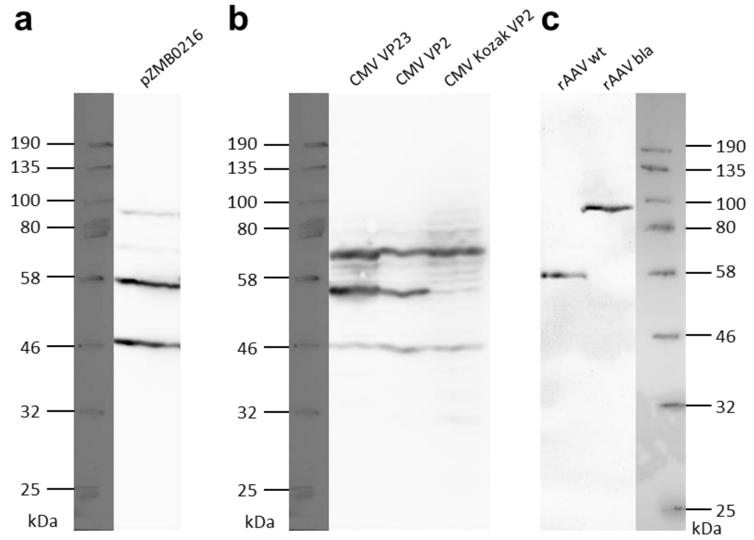

Figure S5. Full length western blot images. **a)** Western blot of crude cell lysate after HEK293 triple-transfection with pZMB0216, pZMB0522 and the pHelper plasmid. VP proteins were detected with the B1 antibody (Progen), a secondary HRP-coupled antibody and subsequent chemiluminescence imaging for 270 s. **b)** Western blot of crude cell lysate after transfection with the above-mentioned plasmids. VP proteins were detected with the B1 antibody (Progen), a secondary HRP-coupled antibody and subsequent chemiluminescence imaging for 200 s. **c)** Western blot of a rAAV wt and rAAV bla from purified viral stocks. VP proteins were detected with the B1 antibody (Progen), a secondary HRP-coupled antibody and subsequent chemiluminescence imaging for 600 s.

2.6 Figure S6

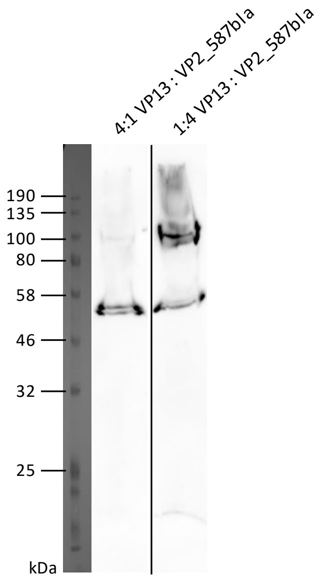

Figure S6. Western Blot analysis for rAAV2_mosaicVP2_587bla with respect to the gene dose of each plasmid. Crude cell lysate was applied to a 10 % SDS PAGE gel for electrophoresis. After semi-dry blotting onto a nitrocellulose membrane the VP proteins were detected with the B1 antibody (Progen). The plasmid ratio of the quadruple transfection was based on the ratio of the triple transfection. ITR, RC and pHelper plasmid have been used in a molar ratio of 1:1:1. To create a mosaic virus, CMV_Kozak_VP2_587bla and Rep2Cap2_VP13 have been transfected. The ratio of these plasmids was varied between 4:1 and 1:4.

2.7 Figure S7

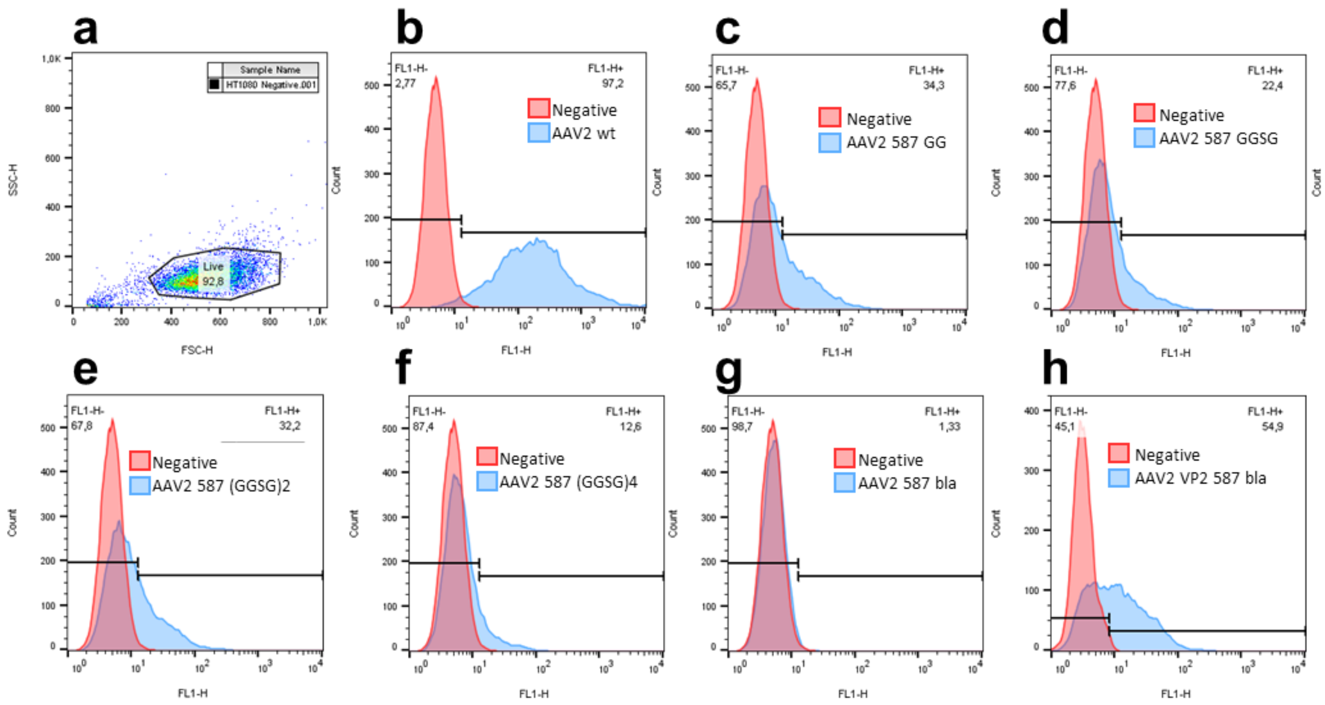

Figure S7. Flow cytometry data for rAAV variants in comparison the negative buffer control. HT1080 cells were transduced with rAAVs at a MOI of 50,000 and analyzed via flow cytometry after incubation. Data analysis was performed using FlowJo. In a first step a population of live cells was gated (**a**). In a second step a gate of 1% false positive cells was selected in the sample of the negative control. This gate is visualized in each diagram. Overlay histograms for the wild-type rAAV2 **b**), all glycine-serine linker variants **c-f**) and both bla variants **g-h**) are shown.

2.8 Figure S8

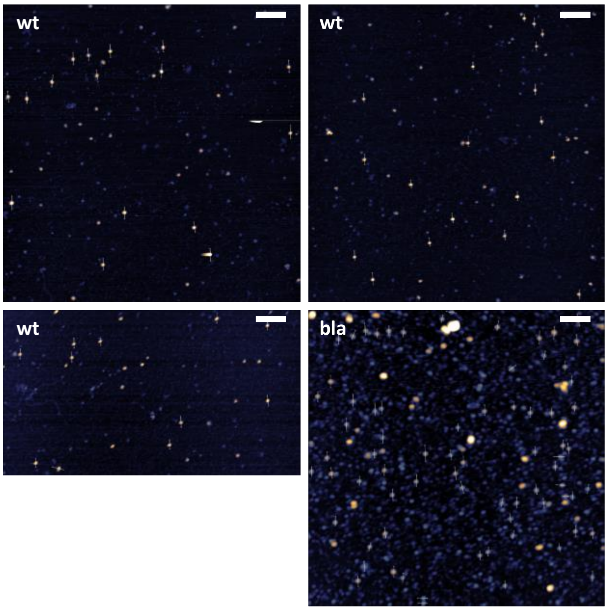

Figure S8. Atomic force micrographs of wild-type (wt) and fully β-lactamase modified rAAV (bla). The scale bars indicate 200 nm. Thin white lines with ticks show height profiles that were extracted to measure the individual particle diameter for particles that met a height threshold of 5 nm (wild-type capsids) and 6 nm respectively. Particle width was determined at half maximum height.

2.9 Figure S9

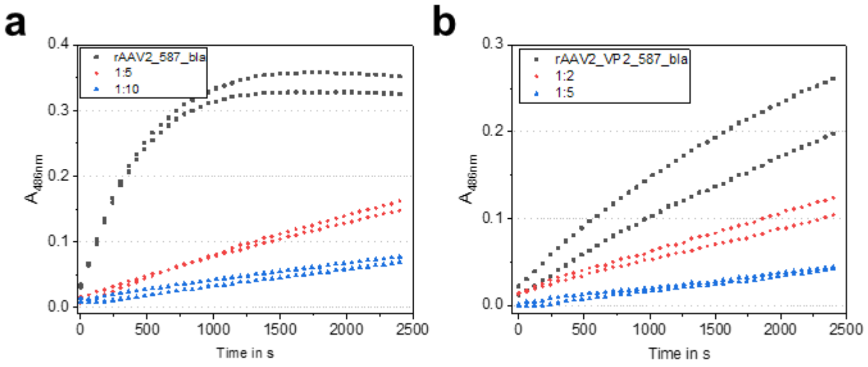

c

$$\frac{\Delta A}{\Delta t} = \Delta\varepsilon_{486} \cdot c_{bla} \cdot k_{cat_{bla}} \cdot d$$

Sample	Slope in s^{-1}	c(bla) in mol/L	c(rAAV) in mol/L	k_{Cat} in s^{-1}
AAV bla full	5.26×10^{-4}	1.65×10^{-10}	6.14×10^{-12}	334.3
AAV bla full 1:5	7.31×10^{-5}	2.29×10^{-11}	1.23×10^{-12}	232.1
AAV bla full 1:10	2.21×10^{-5}	6.95×10^{-12}	6.14×10^{-13}	140.6
AAV bla mosaic	1.22×10^{-4}	3.83×10^{-11}	6.14×10^{-12}	930.7
AAV bla mosaic 1:2	5.50×10^{-5}	1.73×10^{-11}	3.07×10^{-12}	838.2
AAV bla mosaic 1:5	1.88×10^{-5}	5.90×10^{-12}	1.23×10^{-12}	716.6

Figure S9. Nitrocefin assay for determination of β-lactamase activity. **a, b)** Absorption measurements of rAAV2_587_bla and rAAV2_VP2_587_bla in different concentrations at 486 nm. Data points were measured in 1 min intervals. **c)** The first six data point were included in linear regression calculations. Resulting values are given in the table and correlate with dilution. Since very low concentrations were used, there are variations which can be explained in the pipetting the dilutions.

3 Supplementary Information—Tables

3.1 Table S1

Table S1. Overview of plasmids constructed by the iGEM Freiburg team in 2010 including a short description of their features.

Plasmid name with description	Length	Backbone
**pZMB0246_CMV_VP1up_NLS_mVenus_VP23_453_587koHis** expression of VP1 with N-terminal mVenus as well as VP2 and VP3, R585 and R588 in 587 loop regions are changed to Ala (HSPGko), His-tag in 587 loop	5863 bp	pSB1C3_001
**pZMB0091_CMV_DARPinE01_mli_VP23 _453_587koHis** expression of VP2 with N-terminal DARPin E01 fusion, Arg in 587 loop changed to Ala (HSPGko)	5191 bp	pSB1C3_001
**pZMB0156_VP23_453_587wt** construct for cloning, encodes VP2/3 with cloning ready loop regions, allows for the construction of a N-terminal VP2 fusion	4004 bp	pSB1C3_001 RFC[25]
**pZMB0503_VP1up_NLS** construct for cloning, encodes the unique upstream region of VP1 (VP1up) and a nuclear localization sequence (NLS), precursor for assembly of surface exposed VP1 integrations	2525 bp	pSB1C3_001 RFC[25]
**pZMB0143_CMV** CMV promoter in RFC[10] for construction of N-terminal VP2 fusion vectors or ITR plasmid assembly	2725 bp	pSB1C3 RFC[10]
**pZMB0135_hGHpA** hGH polyadenylation signal in RFC[10] for assembling of the ITR plasmid.	2550 bp	pSB1C3 RFC[10]

3.2 Table S2

Table S2. Transduction ability of mosaic and full modified rAAV variants using varying MOIs.[a,b]

MOI	Transduction ability in %			
	**rAAV2_VP2_587_(GGSG)4**	**rAAV2_587_(GGSG)4**	**rAAV2_VP2_587_bla**	**rAAV2_587_bla**
**10**	1.1 ± 0.2	n. d.	1.1 ± 0.1	n. d.
**100**	2.4 ± 0.2.	n. d.	1.8 ± 0.4	n. d.
**1000**	17.2 ± 0.9	0.8 ± 0.1	12.9 ± 1.7	1.1 ± 0.2
**5000**	55.9 ± 0.3	1.7 ± 0.0	46.7 ± 0.5	1.9 ± 0.1
**10000**	71.2 ± 1.2	2.2 ± 0.1	63.4 ± 0.1	4.1 ± 0.6

[a] Transduction ability was assayed with flow cytometry of HT1080 cells and is given as percentage of mVenus expressing cells. The error is based on biological duplicates from one viral preparation. Note that 1% is the background threshold.

[b] The data are based on viral preparations independently produced from the particles in the main text as well as on newly cultivated cells.

3.3 Table S3

Table S3. Characteristics of TEM β-lactamase variant 14FM.[a]

Property	β-lactamase 14FM
Mutations compared to wild-type TEM-116 β-lactamase (GenBank ID: AY425988)	V31A, A36L, L51I, R120G, E147G, H153R, V159T, M182T, L201P, I208M, E212K, A224V, A249V, T264M
Melting temperature T_M determined using far-UV circular dichroism in phosphate buffer	71.6 °C (2 μM)<br>72.7 °C (20 μM)
Catalytic constant k_{cat} for nitrocefin conversion	746 ± 76 s^{-1}

[a]Source: T. Baumann, 2013, PhD thesis, University of Potsdam, Germany, "Stability and interconnected protein properties studied with TEM β-lactamase"

4 Cloning strategy

4.1 Cloning of loop modifications in pZMB0216

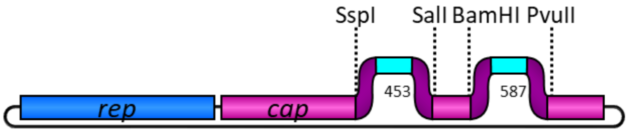


```
Residue 453 (position determined via Uniprot #P03135)
For 5′-ATTTGTATTACTTGAGCAGAACAAACACTCCAAGTGGTNNN…NNNACCACCACGCAGAG-3′
Rev 5′-TAAACATAATGAACTCGTCTTGTTTGTGAGGTTCACCANNN…NNNTGGTGGTGCGTCTCAGCT-3′

Residue 587 WT
For 5′-GATCCGTATCTACCAACCTCCAGAGAGGCAACNNN…NNNAGACAAGCAGCTACAG-3′
Rev 5′-GCATAGATGGTTGGAGGTCTCTCCGTTGNNN…NNNTCTGTTCGTCGATGTC-3′

Residue 587 KO
For 5′-GATCCGTATCTACCAACCTCCAGGCTGGCAACNNN…NNNGCCCAAGCAGCTACAG-3′
Rev 5′-GCATAGATGGTTGGAGGTCCGACCGTTGNNN…NNNCGGGTTCGTCGATGTC-3′

Viral Brick 587ko-empty
For 5′-GATCCGTATCTACCAACCTCCAGGCTGGCAACGCCCAAGCAGCTACAG-3′
Rev 5′-CTGTAGCTGCTTGGGCGTTGCCAGCCTGGAGGTTGGTAGATACG-3′

Viral Brick 587-GG
For 5′-GATCCGTATCTACCAACCTCCAGAGAGGCAACGGAGGCAGACAAGCAGCTACAG-3′
Rev 5′-CTGTAGCTGCTTGTCTGCCTCCGTTGCCTCTCTGGAGGTTGGTAGATACG-3′

Viral Brick 587-GGSG
For 5′-GATCCGTATCTACCAACCTCCAGAGAGGCAACGGAGGCTCTGGTAGACAAGCAGCTACAG-3′
Rev 5′-CTGTAGCTGCTTGTCTACCAGAGCCTCCGTTGCCTCTCTGGAGGTTGGTAGATACG-3′

Viral Brick 587-2x(GGSG)
For 5′-GATCCGTATCTACCAACCTCCAGAGAGGCAACGGAGGCTCTGGTGGCGGTTCAGGAAGACAAGCAGCTACAG-3′
Rev 5′-CTGTAGCTGCTTGTCTTCCTGAACCGCCACCAGAGCCTCCGTTGCCTCTCTGGAGGTTGGTAGATACG-3′

Viral Brick 587-4x(GGSG)
For  5′-GATCCGTATCTACCAACCTCCAGAGAGGCAACGGAGGCTCTGGTGGCGGTTCAGGAGGTGGAAGCGGCGGAGGTAGTGGCAGACAAGCAGCTACAG-
3′
Rev 5′-CTGTAGCTGCTTGTCTGCCACTACCTCCGCCGCTTCCACCTCCTGAACCGCCACCAGAGCCTCCGTTGCCTCTCTGGAGGTTGGTAGATACG-3′
```

Figure S11. Cloning strategy for 453 and 587 loop regions. Sequences of oligonucleotides used for the generation of different loop modifications by hybridization are shown. The given oligonucleotides have been cloned via the above mentioned restriction enzymes.

4.2 Cloning of N-terminal VP2 modifications for rAAV2

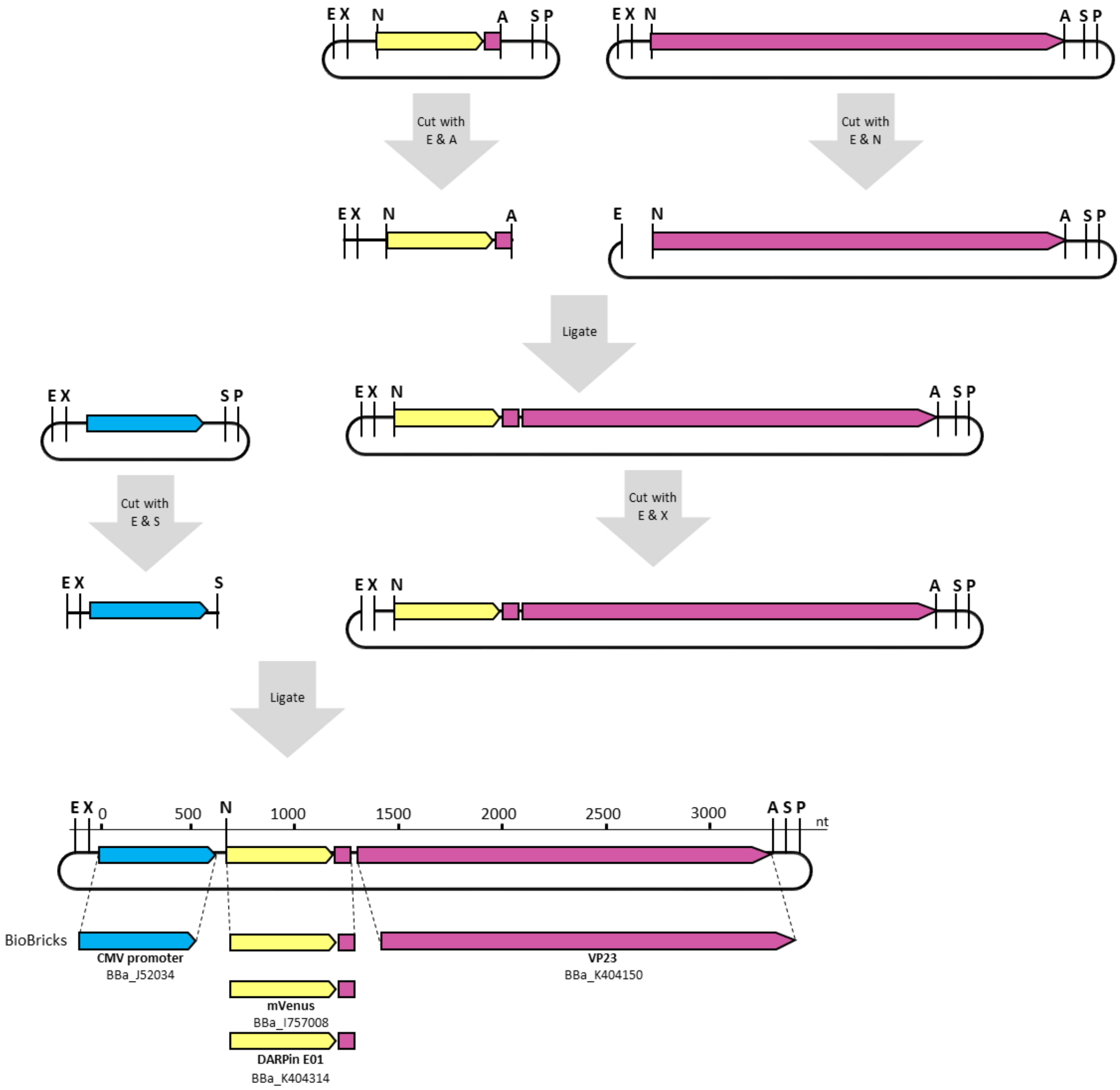

Figure S12. Cloning strategy for pZMB0091_CMV_GOI_mli_VP23 _453_587koHis. This plasmid was not used during this work but shows the potential assembly of N-terminal fusion proteins. Assembly of BioBricks can be performed as shown. The following abbreviations are used for the restriction enzymes and their recognition sites, respectively: E= EcoRI, X= XbaI, N= NgoMIV, A= AgeI, S= SpeI, P= PstI.
